# The Research of Tool Wear Mechanism for High-Speed Milling ADC12 Aluminum Alloy Considering the Cutting Force Effect

**DOI:** 10.3390/ma14051054

**Published:** 2021-02-24

**Authors:** Xinxin Meng, Youxi Lin, Shaowei Mi

**Affiliations:** School of Mechanical Engineering and Automation, Fuzhou University, Wulongjiangbei Avenue Road 2 of Fuzhou University City, Fuzhou 350108, Fujian, China; m170210003@fzu.edu.cn (X.M.); mswfzu123@163.com (S.M.)

**Keywords:** ADC12 aluminum alloy, high-speed milling, cutting force, tool wear

## Abstract

Tool wear is a major cause of accelerated tool failure during the milling of aluminum alloy. The periodically cutting force directly affect the cutting heat and tool wear due to the intermittent cutting characteristics of the milling process. The focus of this paper is to analyze the influence of the variation of cutting force on tool wear behavior. The change law of cutting force by cutting parameters was analyzed firstly. Secondly, the variation of the wear land width (VB) of tool flank face by the milling length was analyzed. Thirdly, the wear morphology and the energy dispersive spectrometer (EDS) results of tool rake face and flank face in different cutting parameters were observed by tungsten filament scanning electron microscope. Finally, considering the cutting force effect, the tool wear mechanism during high-speed milling of Aluminum-Alloy Die Castings 12 (ADC12, 12 means aluminum number 12) was analyzed. The cutting force in tangential direction is predominant during high-speed milling aluminum alloy, which decreases gradually with the increase of cutting speed but increases gradually with the feed rising. The adhesion-oxidation wear was main wear mechanism of tool rake face during high-speed milling. While adhesive wear was the main wear mechanism of the tool flank face during high-speed milling. It is found that the formation of adhesive wear is the process from particle adhesion to melting until the formation of adhesive layer, which related to the change of cutting force.

## 1. Introduction

With the development of the automobile industry, ADC12 aluminum alloy has been widely used in an engine cylinder body and head with its low density, good casting performance, wear resistance, and small thermal expansion coefficient [1]. Due to the existence of silicon-containing crystals in this alloy, serious tool wear, high cutting heat were easy to generate during high-speed cutting, which will shorten tool life, increase workpiece deformation and reduce productivity [2]. In addition, the periodically varying cutting force directly affect the cutting heat, further affecting tool wear and machining surface quality because the milling process is a multi-tooth discontinuous cutting process. The workpiece and tool undergo a periodic load-unload process at each tooth cycle. Cyclic load causes cyclic mechanical force and thermal stress on the tool [3,4]. Therefore, it is necessary to analyze the influence of the variation of cutting force on tool wear during high-speed milling of ADC12 alloy. In addition to the machine tool, tool geometry parameters, the cutting fluid and cutting parameters also have impact on the cutting force during metal cutting. Among them, the cutting parameters have the greatest influence. The cutting speed has the greatest influence on the cutting force, followed by the feed, and the last by cutting depth [4]. The cutting temperature also has an effect on the cutting force. Especially the tool-chip interface temperature, which affects the distribution of cutting force, tool wear morphology and machining surface quality. The maximum cutting temperature in cutting zone rising exponentially as cutting speed increases [5,6,7]. Feng et al. [8] proposed a new prediction model of cutting temperature considering the effect of dynamic recrystallization. Cui et al. [9] analyzed the influence of chip temperature on chip morphology. The higher the temperature, the more ductile the chip. The higher chip temperature caused lower cutting force and more stable cutting process, which is conducive to better surface finish.

To research the effect of cutting force and tool rotation radius on the tool wear, Alessandro Colpani et al. [10] proposed a statistical analysis method based on Pearson correlation coefficient, and illustrated that the tool flank wear can be used for a criterion of tool life. The minimum chip thickness significantly influences the cutting force, tool wear, and machining stability of machining process, the prediction of that is very important to study cutting force and tool wear [11].

An accurate prediction model of tool wear is essential to analyze the tool wear process [12]. Considering the thermodynamic coupling effect during milling, tool wear model can be constructed using Fick diffusion law [13]. M. Pradeep Kumar [14] researched the effect of machining variables on tool life and various tool failure mechanisms. Considering the impact of tool wear on the machined surface, the surface morphology during cutting according to the tool wear morphology and the additional thermal and mechanical load were studied, and the plastic flow, surface burn, scratch and other undesirable surface defects are discussed. The experimental results show surface morphology and tool wear are positively correlated with each other [15]. Tool material and coating material also have the influence on the variation of cutting force and the distribution of cutting temperature, thus affecting the tool wear mechanism [16,17]. Aluminum titanium nitride (TiAlN) coated tools have the highest wear resistance at the cutting speed of 30 m/min [18]. The larger fillet feed radius can lower the tool wear significantly by comparing the milling experiments of different fillet feeding methods [19].

Wang et al. [20] believed that the cutting mechanism changed back and forth between the ploughing and shearing during milling. The ratio of feed per tooth to tool fillet radius (f/r) has an important influence on the milling mechanism [21]. The surface quality has been improved using the up milling comparing with the down milling [22]. In addition, many scholars have studied the relationship between the cutting force and tool wear of laser assisted milling and ultrasonic vibration assisted milling [23,24,25]. It found that adhesive wear is the main wear mechanism of tool failure. The adhesion and diffusion are most important causes of tool wear in dry machining of aluminum alloy with cemented carbide insert [26]. Adhesion arising from workpiece material pick upon the rake face and partial melting of the chip due to the high temperatures and pressure conditions on the tool edge. Diffusion wear of tool-chip occurs when the temperature is greater than a certain critical temperature. Oxidative wear mainly exists in high-speed cutting of aluminum alloy [27,28,29,30]. This is mainly determined by the mechanical properties of aluminum alloy. The finite element method is mainly used to study the influence of material mechanical properties and cutting temperature on the tool wear behavior [31,32,33].

In conclusion, most researchers only study the effect of cutting parameters on cutting force and cutting temperature, or only study the tool wear behavior under different cutting parameters. The innovation of this paper is to analyze the influence of the variation of cutting force on tool wear behavior. The key point is the relationship between cutting force and tool wear. To solve the problem, firstly the changing law of cutting force with the cutting parameters was analyzed. Secondly the tool wear behavior at different cutting parameters has been studied, which included the variation of wear land width (VB), the wear morphology of rake face and flank face of the tool, and the EDS results of wear morphology of tool rake face and flank face. Finally, the tool wear mechanism was analyzed considering the cutting force effect. It is found that the formation process of adhesive layer on rake face is the process from particle adhesion to melting until the formation of adhesive layer. The change of adhesion particle shape is related to the variation of cutting force.

## 2. Experiment Details

### 2.1. Experimental Equipment and Carbide Tool

**Figure 1:**
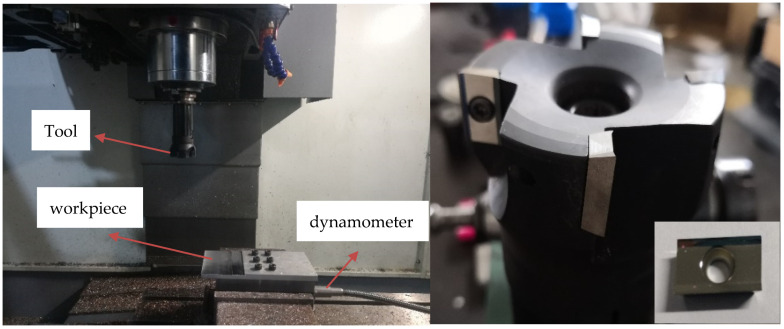
Experimental setup for high-speed milling.

The milling experiments were conducted for ADC12 aluminum alloy to study the variation of milling force at different cutting parameters. Table 1 lists the chemical composition of the alloy. The machine tool used in the test was vmc-850e of vertical machining center, with the maximum spindle speed of 8000 rpm and the maximum rated current of the motor 95 A. All the milling tests were conducted without cutting fluid. The hilt is BT40 series (Where BT represents the tool hilt with a taper of 7/24, and 40 represents the specification, matching the spindle of the machine tool), the cutting head is 400r-63-22-4t, the diameter of the cutting tool is 63 mm, and it can carry 4 inserts. The insert is apkt1604pdfr-ma H01 series computer numerical control (CNC) milling blade for aluminum from Korea Chloe company. The physical properties of the alloy and carbide inserts are shown in Table 2. The cutting parameters of single-factor milling experiment are shown in Table 3. The insert is fine grain carbide with good wear resistance and toughness, suitable for fine and semi-finish processing of aluminum and other non-ferrous metals. The workpiece size is 200 × 100 × 10 mm^3^. The cutting forces in three directions during milling were measured by Kistler dynamometer. Experimental Settings are shown in Figure 1.

### 2.2. Detailed Experimental Scheme

The Kistler piezoelectric tri-directional dynamometer (type 9257B) was installed on the machine tool, fixed with a fixture, and the workpiece was fixed on the dynamometer with screws. Three repeated milling force measurement tests were performed for each cutting parameter. The cutting force under the last three milling paths was measured when the milling length reached 2.5 m. The corresponding tool wear morphology was observed by Phenom desk scanning electron microscope when the milling length reached 2.5 m. By comparing three groups of cutting force data, the most stable data was selected as the cutting force under the cutting parameters. The VB can be obtained by measured the wear land width of the flank face of the tool. This measurement method does not consider the influence of distortion, which basically does not affect the measurement value of VB. The tool wear mechanism and the distribution characteristics of chemical elements on the tool rake face were analyzed by the tungsten filament scanning electron microscope (SEM) with energy spectrometer (EDS) when the milling length reached 20 m.

## 3. Results and Discussion

### 3.1. Variation of the Cutting Force in High-Speed Milling

High-speed milling process is a periodic intermittent cutting process. The cutting force changes periodically as cutting thickness during milling. As the cutting edge periodically enters and exits the workpiece, the tool undergoes stress and temperature cycles during the cutting process.

**Figure 2:**
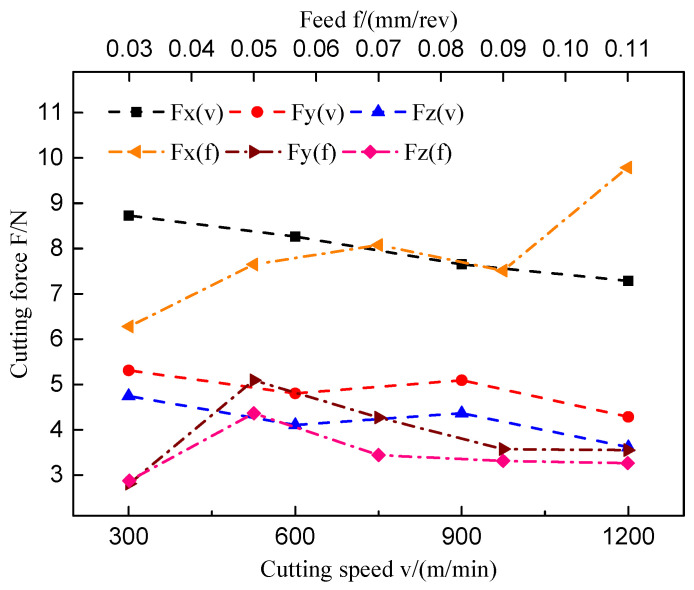
The variation of cutting force by the cutting parameters(The feed is 0.05 mm/rev, cutting depth is 0.5 mm and cutting width is 3 mm when the cutting speed changes; The cutting speed is 900 m/min, cutting depth is 0.5 mm and cutting width is 3 mm when the feed changes).

The cutting force components are divided into tangential force, radial force and axial force in the milling process, which measured by the Kistler dynamometer are Fx, Fy, and Fz respectively. Figure 2 shows the variation of cutting force by the cutting parameters. When studying the effect of cutting speed on the cutting force, the feed and cut depth are 0.05 mm/rev and 0.5 mm respectively, and the cutting width is 3 mm. When studying the effect of feed on cutting force, the cutting speed and cutting depth are 900 m/min and 0.5 mm respectively, and the cutting width is 3 mm. It shows that the tangential cutting force Fx predominated comparing with the radial force and axial force. With the cutting speed increasing, tangential cutting force decreased gradually. With the feed per revolution increasing, tangential cutting force rising gradually.

### 3.2. Variation of the Wear Land Width of Flank Face

**Figure 3:**
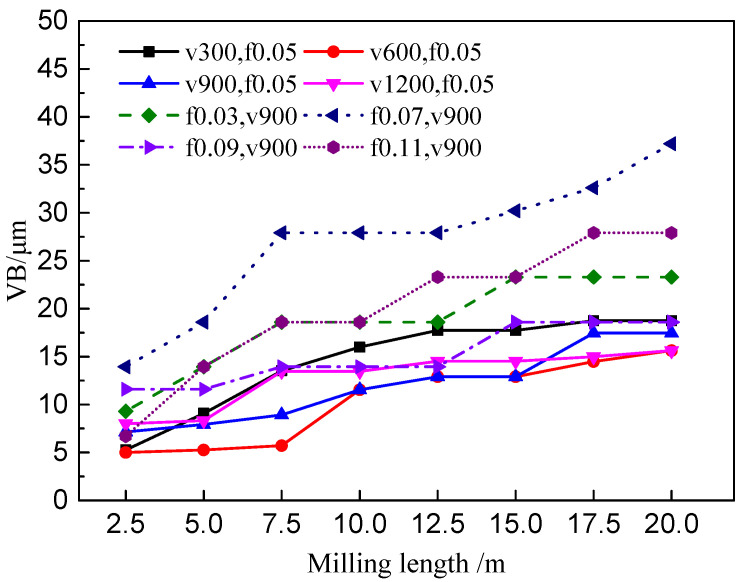
The change curve of VB with milling length at different cutting parameters (where V means cutting speed in m/min, F means the Feed in mm/rev, the cutting depth is 0.5 mm and cutting width is 3 mm).

To analyze the tool wear mechanism, the variation of tool wear land width (VB) during high- speed milling of ADC12 alloy has been studied firstly. By observe the wear morphology of tool flank face in different milling length, the change curve of wear land width by milling length at different cutting speed and feed can be obtained as shown in Figure 3. It shows that the tool maximum wear land width reached about 20 μm at 300 m/min when milling length reached 20 m. With the cutting speed rising, the wear land width decreasing gradually except for 600 m/min. When the milling length reached 20 m, the wear land width of flank face is the largest at 0.07 mm/rev, which reached about 40 μm, followed by 0.11 mm/rev and 0.03 mm/rev. The wear land width of flank face is the smallest at 0.09 mm/rev.

### 3.3. The Tool Wear Morphology

#### 3.3.1. The Tool Wear Morphology of Rake Face

**Figure 4:**
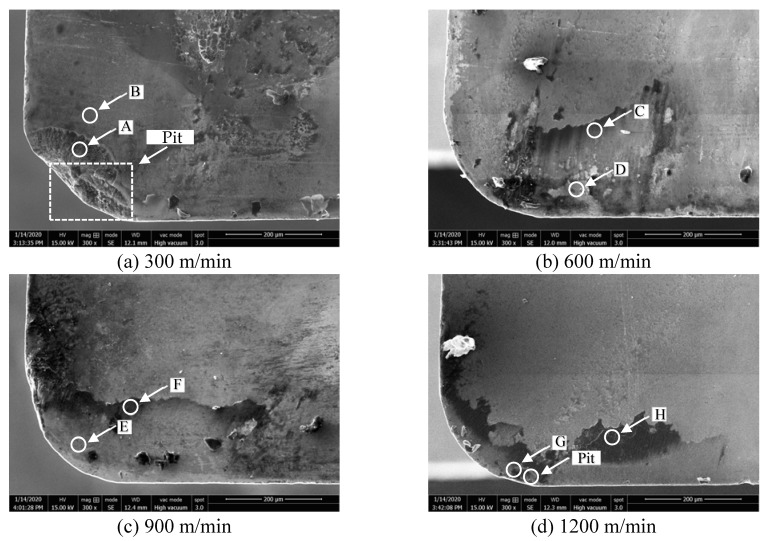
The wear morphology on rake face at different cutting speed (0.05 mm/rev, cutting depth 0.5 mm, cutting width 3 mm). (**a**)300 m/min; (**b**) 600 m/min; (**c**) 900 m/min; (**d**) 1200 m/min.

**Figure 5:**
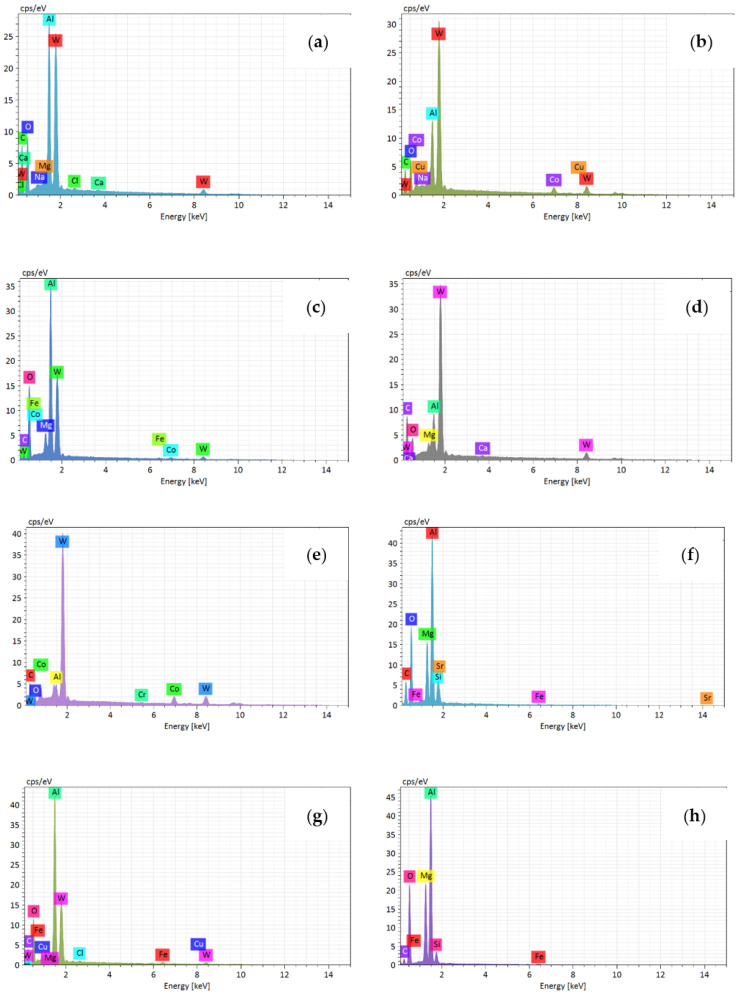
The energy disperse spectroscopy results of rake face at different cutting speed ((**a**–**h**) represent the points A~H in the Figure 4 respectively).

In order to analyze the wear mechanism of the tool rake face, the chemical element distribution on the rake face was analyzed by means of tungsten filament scanning electron microscope. Figure 4a–d shows the wear morphology of rake face when the milling length reached 20 m at different cutting speed. It illustrates that the severe pit wear and adhesion were generated on the rake face at 300 m/min. Only the adhesion was generated on the rake face at other cutting speeds, which includes the adhesion of a single hard particle and the adhesive layer of a molten state. To analyze the wear mechanism of rake face at different cutting speeds, the points in the adhered zone and the unadhered zone were selected, and the EDS results of each point in the Figure 4a–d are shown in Figure 5a–h, respectively.

Figure 4a shows that pit wear and adhesion were generated on the tool rake face at 300 m/min. Therefore, point A in the pit and point B on the tool face are selected, and their corresponding EDS spectrum analysis is shown in Figure 5a,b. It can be seen that point A is mainly aluminum and tungsten, in addition to the presence of oxygen and magnesium. Point B is mainly tungsten, with aluminum and cobalt. It illustrates that a small amount of adhered aluminum was generated on the pits at 300 m/min. This is due to the friction between the cutting tool and the chip during milling, the temperature gradually increases, the chip accumulated and adhered to the rake face, so that the aluminum element of the chip appeared on the tool rake face. Meanwhile, the high content of oxygen element and the appearance of magnesium indicate that oxidation reaction occurs and oxides are generated. Therefore, the pit wear and the adhesion wear were the main wear mechanism of the tool rake face at 300 m/min, and the oxidation wear was also present.

Figure 4b shows that severe adhesion was generated on the rake face at 600 m/min. As can be seen from the Figure 5c,d, point C in the adhesion zone is mainly aluminum and oxygen. Point D in the unadhered zone is dominated by tungsten, while oxygen, aluminum and magnesium are also present. This indicates that adhesive wear was generated on the rake face at 600 m/min. In addition, the presence of oxygen, iron, and magnesium indicates the oxidative wear was also present.

At 900 m/min, it is found that the tungsten element is the main element on the rake face at the unadhered zone. Adhesion zone is dominated by the aluminum element, followed by oxygen element and magnesium element, indicating that oxidative wear is also produced at tool rake face besides adhesive wear. Figure 4d shows that there are also pits on the rake face in addition to surface adhesion at 1200 m/min. There is also a lot of adhesive aluminum element in pit G, and others mainly tungsten element of tool matrix. Adhesion zone point H is dominated by aluminum, oxygen and magnesium, with small amounts of iron and silicon. This indicates that the adhesive wear and oxidation wear were the main wear mechanism of the rake face at 1200 m/min.

In summary, the wear mechanism of the tool rake face at different cutting speed is basically the same, which is dominated by adhesive wear and oxidation wear. What is more, pit wear occurs on the tool rake face at 300 m/min and 1200 m/min.

**Figure 6:**
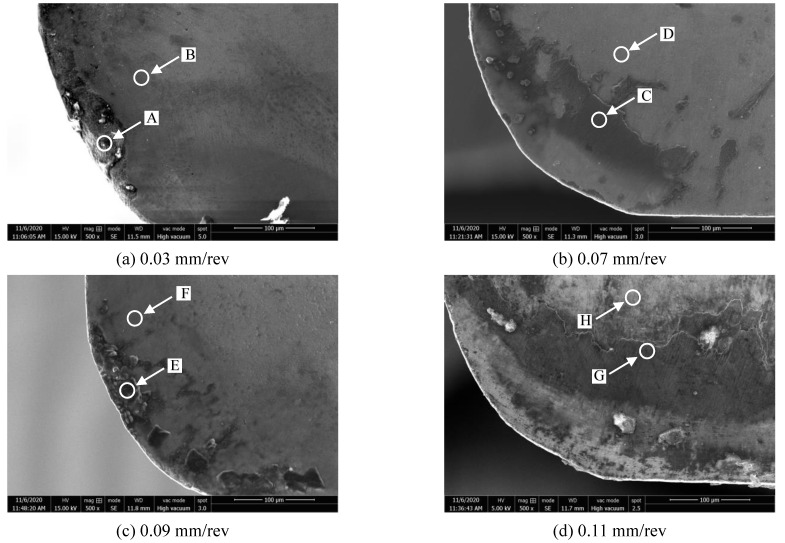
The wear morphology of rake face at different feed (cutting speed 900 m/min, cutting depth 0.5 mm, cutting width 3 mm). (**a**) 0.03 mm/rev; (**b**) 0.07 mm/rev; (**c**) 0.09 mm/rev; (**d**) 0.11 mm/rev.

Figure 6a–d shows the wear morphology of tool rake face at the feed per revolution of 0.03 mm/rev, 0.07 mm/rev, 0.09 mm/rev, and 0.11 mm/rev when the milling length reached 20 m, respectively. It shows that the pit wear was also generated on the rake face at the feed rate of 0.03 mm/rev. Adhesion was the main wear morphology of rake face at other feeds.

**Figure 7:**
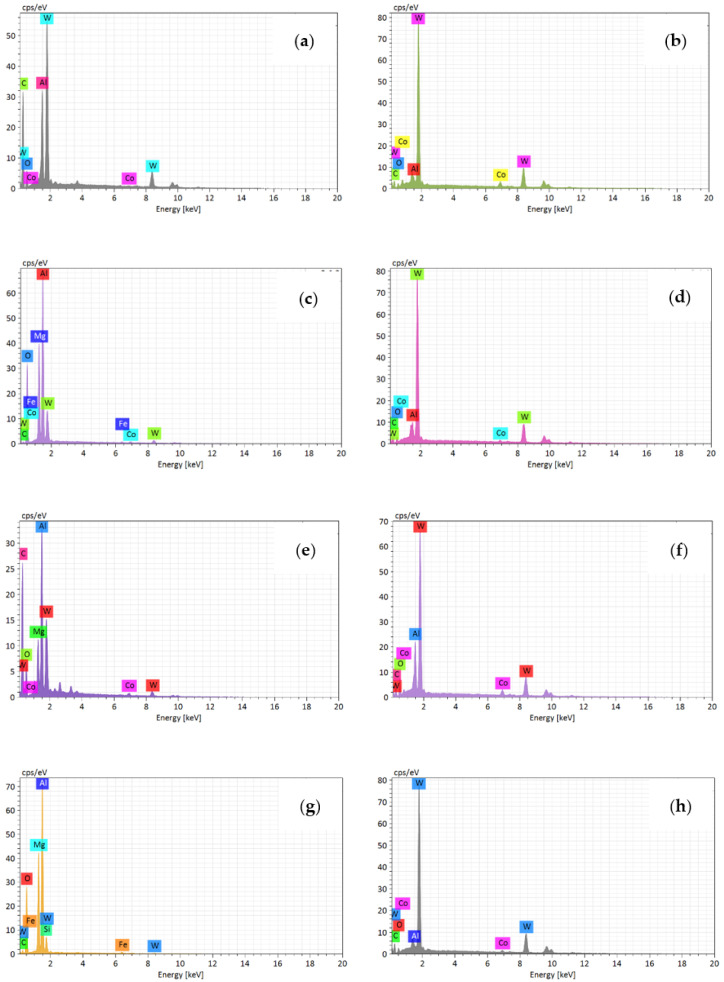
The energy dispersive spectrometer (EDS) results of rake face wear morphology at different feed ((**a**–**h**) represent the points A~H in the Figure 6 respectively).

The corresponding EDS energy spectrum results of the points on the rake face of Figure 6a–d was shown in Figure 7a–h respectively. It shows that the point A of the pit on the rake face mainly composed of tungsten element of the tool matrix and aluminum element at the same time. The point B is mainly the tungsten element of the tool matrix. The illustrates that the pit wear and the adhesion wear were generated at the same time. The point C in the adhesion zone of rake face is mainly aluminum, followed by magnesium and oxygen, and iron appears at the same time. The unadhered zone point D is mainly tungsten element of the tool matrix. Therefore, the adhesion wear and oxidation wear were the main wear mechanism of the rake face at 0.07 mm/rev.

It can be seen that the point E of adhesion zone is mainly aluminum, magnesium and oxygen elements. The point F is mainly tungsten element of the tool matrix. So, the adhesive wear was the main wear mechanism of rake face at 0.09 mm/rev. At 0.11 mm/rev, it is found that the adhesion zone is mainly aluminum, oxygen and magnesium, and iron appears at the same time. The unadhered zone is mainly tungsten element of the tool matrix. Therefore, the adhesion wear and oxidation wear were the main wear mechanism of the rake face at 0.11 mm/rev. In summary, it is found that the adhesive wear and oxidation wear were the main wear mechanism of rake face at different feeds when the milling length reached 20 m.

#### 3.3.2. The Wear Morphology of Flank Face

**Figure 8:**
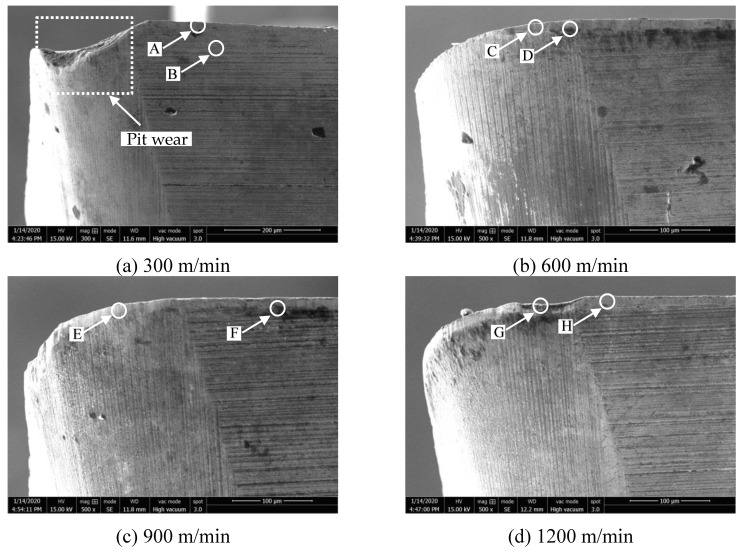
The wear morphology of flank face after milling at different cutting speed (where feed is 0.05 mm/rev, cutting depth is 0.5 mm and cutting width is 3 mm). (**a**) 300 m/min; (**b**) 600 m/min; (**c**) 900 m/min; (**d**) 1200 m/min.

**Figure 9:**
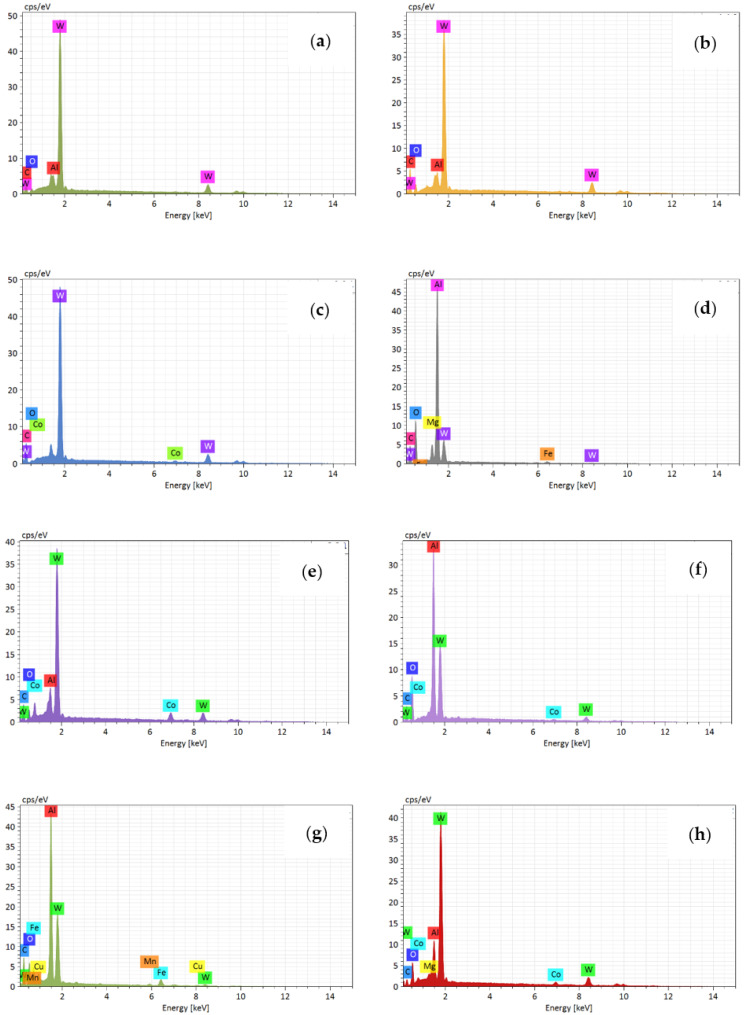
The EDS results of flank face wear morphology at different cutting speed ((**a**–**h**) represent the points A~H in the Figure 8 respectively).

In the previous section, the wear morphology of the tool rake face under different cutting parameters after milling was analyzed. In this section, the wear morphology of the tool flank face under different cutting parameters after milling was analyzed. Figure 8a–d shows the wear morphology of the flank face at the cutting speed of 300~1200 m/min after milling. It can be seen from the figure that severe pit wear appears on the flank face at 300 m/min. Point A of the wear zone and point B of the unwear zone were selected, and the corresponding EDS results were shown in Figure 9a,b. It shows that point A is mainly tungsten element, and point B is also dominated by tungsten, with a small amount of aluminum. This indicates that the pit wear and adhesion wear were the main wear mechanism of the flank face at 300 m/min.

Figure 9c,d shows that point C is dominated by tungsten element and point D is dominated by aluminum element, with oxygen element and magnesium element. Therefore, the slight adhesive wear was generated on the flank face at 900 m/min. Figure 9e,f illustrates that point E is mainly composed of tungsten element of the tool matrix, with a small amount of aluminum element and oxygen element. Point F is mainly aluminum element, with a small amount of oxygen and cobalt. Figure 9g,h shows that the pit G is mainly aluminum element, which indicates that the pit wear and adhesion wear were simultaneous occurred on the flank face. The point H is mainly the tungsten element of the tool matrix. In summary, the adhesive wear and a slight oxidative wear were the main wear mechanism of the flank face after milling at different cutting speed.

**Figure 10:**
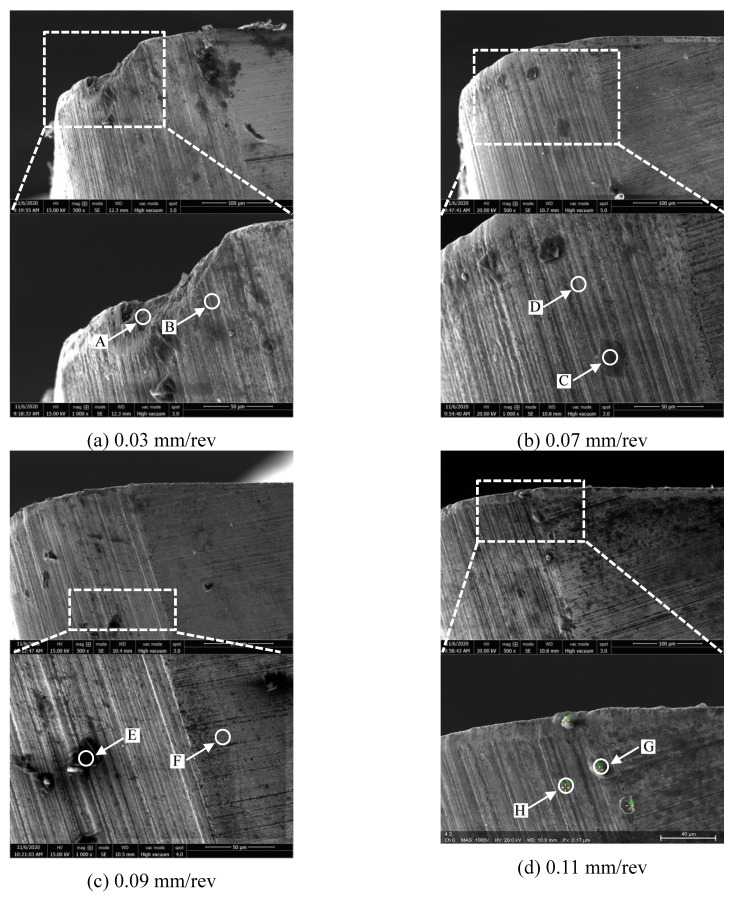
The wear morphology of flank face after milling at different feed (where cutting speed is 900 m/min, cutting depth is 0.5 mm and cutting width is 3 mm). (**a**) 0.03 mm/rev; (**b**) 0.07 mm/rev; (**c**) 0.09 mm/rev; (**d**) 0.11 mm/rev.

**Figure 11:**
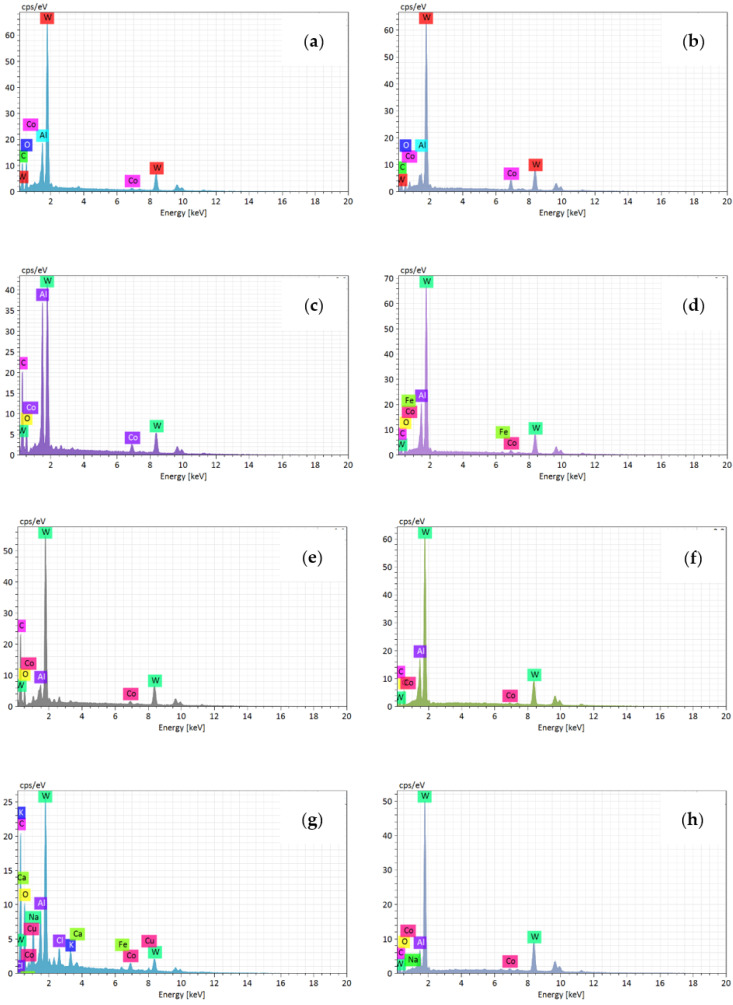
The EDS results of flank face wear morphology at different feed ((**a**–**h**) represent the points A~H in the Figure 10 respectively).

Figure 10a–d shows the wear morphology of the flank face under different feed after milling. The corresponding EDS results of the Points was shown in Figure 11a–h. Figure 10a shows that the severe pit wear was generated on the flank face at 0.03 mm/rev. The EDS results illustrates that the flank face is mainly the tungsten element of the tool matrix, and a small amount of aluminum and oxygen elements.

At 0.07 mm/rev, Figure 11c,d illustrates that the flank face is mainly composed of tungsten element of the tool matrix, aluminum element and oxygen element. Figure 11e,h shows that the tool flank face is also mainly tungsten element of the tool matrix at 0.09 mm/rev and 0.11 mm/rev, which indicates that almost no adhesive wear was generated on the flank face comparing with the wear mechanism of the rake face.

### 3.4. The Tool Wear Mechanism Considering the Cutting Force Effect

The wear morphology and the wear mechanism of the rake face and flank face of the tool under different cutting parameters has been analyzed. The influence of cutting force on the tool wear mechanism during high-speed milling of aluminum alloy was discussed in this part.

**Figure 12:**
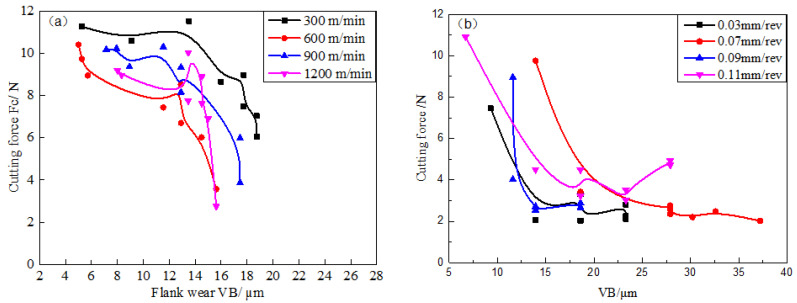
The relationship between cutting force and wear land width (VB) at different (**a**) cutting speed (where feed is 0.05 mm/rev, cutting depth is 0.5 mm and cutting width is 3 mm); (**b**) feed (where cutting speed is 900 m/min, cutting depth is 0.5 mm and cutting width is 3 mm).

Figure 12 shows the relationship between the cutting force and the wear land width (VB) of flank face under different cutting parameters. With the milling length rising, the wear land width of the flank face gradually increases, and the cutting force shows a trend of gradual decline. Figure 12a shows that the cutting force gradually decreases with the increase of cutting speed except for the 600 m/min. Figure 12b shows that the cutting force gradually increases with the increase of feed, except the 0.07 mm/rev. According to the tool wear morphology, it is found that severe adhesion wear was generated on the tool rake face at 600 m/min and 0.07 mm/rev. This means the cutting force has been reduced to a certain extent owing to the adhesive layer.

**Figure 13:**
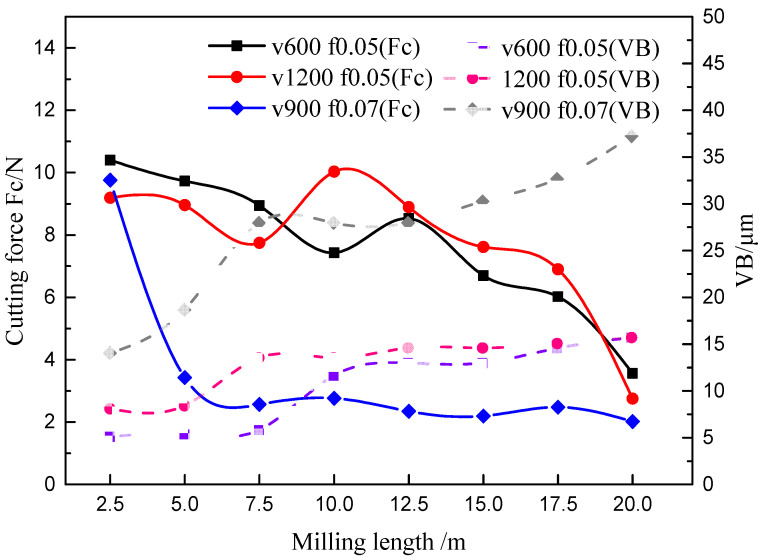
The relationship between the cutting force and VB at different cutting parameters.

**Figure 14:**
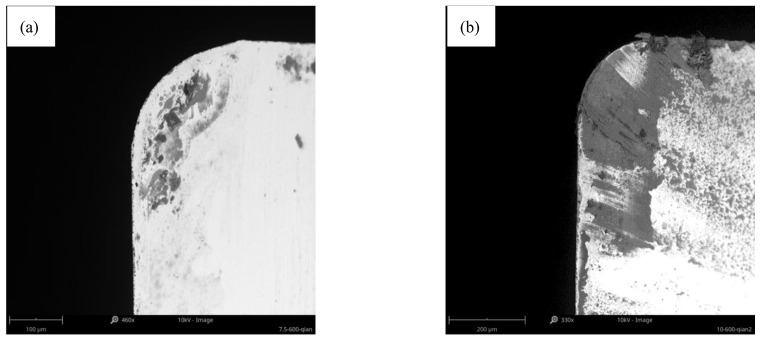
The wear morphology of rake face at 600 m/min when the milling length reached (**a**) 7.5 m; (**b**) 10 m.

So, with 600 m/min, 1200 m/min, and 0.07 mm/rev as an example, the relationship between the cutting force and the tool wear mechanism has been discussed, as shown in Figure 13. It illustrates that the cutting force drops sharply and the wear land width of flank face increases sharply at 600 m/min when the milling length increases from 7.5 m to 10 m. The wear morphology of the rake face at this milling length was observed, as shown in Figure 14a,b. It illustrates that the hard particles adhered to the rake face when milling length reached 7.5 m. But the adhesive layer is formed on the rake face when the milling length reached 10 m. It proves that the cutting force decreases sharply and the VB increases sharply during the transformation from adhesive particle to the adhesive layer. At the same time, the existence of adhesive layer can protect the tool rake face to some extent. So, the pit wear has not been generated on the rake face of the tool at 600 m/min.

**Figure 15:**
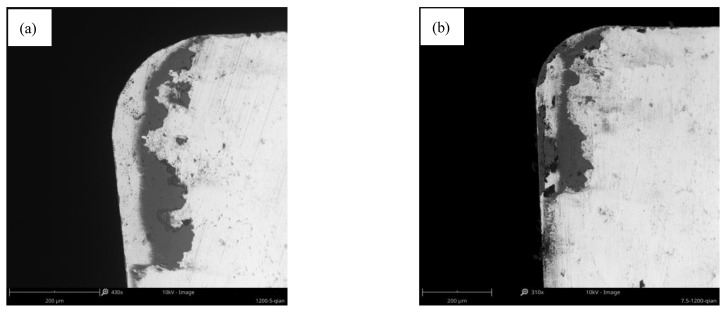
The wear morphology of rake face at 1200 m/min when the milling length reached (**a**) 5 m; (**b**) 7.5 m.

**Figure 16:**
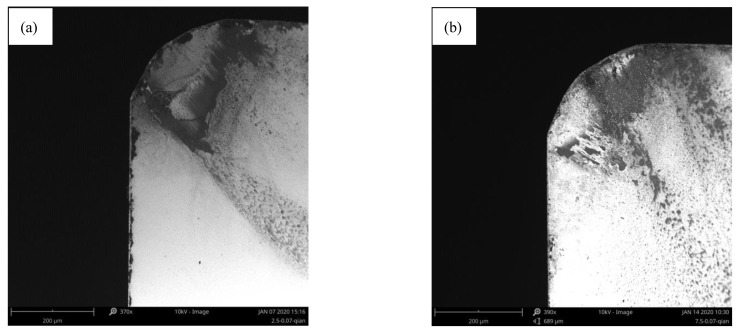
The wear morphology of rake face at 0.07 mm/rev when the milling length reached (**a**) 2.5 m; (**b**)7.5 m.

Similarly, the relationship between the cutting force and the wear land width at 1200 m/min was analyzed. It is found that the cutting force decreases sharply and the VB also increases sharply when the milling length increases from 5 m to 7.5 m. The corresponding wear morphology of the tool rake face is shown in Figure 15a,b. It demonstrates that the adhesion layer increased significantly on the rake face. Figure 13 shows that both cutting force and VB dramatically changed when the milling length increases from 2.5 m to 7.5 m at 0.07 mm/rev. The corresponding wear morphology of tool rake face was shown in Figure 16a,b. It proves that the adhesive particles adhered to the tool rake face when the milling length reaches 2.5 m. Both the molten adhesive layer and hard particles coexist on the tool rake face when the milling length reaches to 7.5 m. This indicates that both the cutting force and the wear land width of flank face changes dramatically during the formation of the adhesive layer. The change rate of cutting force and the change rate of the wear rate of the tool surface tends to be stable when the adhesive layer is formed.

## 4. Conclusions

This work focuses on the tool wear behaviors and corresponding cutting force during high-speed milling ADC12 aluminum alloy with an uncoated carbide tool. The main conclusions are summarized as follows.

(1) The cutting force in tangential direction is predominant during high-speed milling of aluminum alloy, which decreases gradually with the increase of cutting speed but increases gradually with the feed rising. The wear land width (VB) of flank face increases with the cutting speed and feed rising. The adhesion-oxidation wear was main wear mechanism of tool rake face during high-speed milling, while the adhesive wear was the predominant wear mechanism of flank face. The pit wear was generated on the tool surface when the milling length reaches 12.5 m at 300 m/min and 1200 m/min, which reduced tool life.

(2) The formation of adhesive layer on the rake face is from the adhesion of hard particle—particle melting—forming adhesive layer. The formation process of adhesive layer leads to a sharp decrease in cutting force and a sharp increase in the VB of the flank face. The change rate of cutting force and the change rate of the wear rate of the tool surface tends to be stable when the adhesive layer is formed. The adhesive wear of the rake face can be reduced by monitoring the cutting force during high-speed milling of aluminum alloy.

(3) The cutting force and the tool wear morphology were measured after milling per 2.5 m in this paper. The tool cooling due to the interruption of the experiment was ignored, which is the limitation of this study. In this study, it was found that the shape of adhesive layer on the tool rake face changed during high-speed milling. This may be related to the temperature of tool-chip interface during high-speed milling. Later studies focused on the formation mechanism of adhesive wear analyzing from the variation of cutting temperature during high-speed milling of aluminum alloy.

## Figures and Tables

**Table 1:** Compositions of the Aluminum-Alloy Die Castings 12 (ADC12) aluminum alloy.

Chemical Compositions (%)								
Si	Fe	Cu	Mg	Mn	Zn	Ni	Sn	Al
9.6–12	<1.3	1.5–3.5	<0.3	<0.5	<1.0	<0.5	≤0.3	others

**Table 2:** The material parameters of ADC12 aluminum alloy and cemented carbide tool.

Material Parameter	Workpiece	Tool
density/kg·m^−^^3^	2.67 × 10^3^	15 × 10^3^
Young modulus/GPa	76	800
Poisson’s ratio	0.33	0.2
specific heat/J·kg^−^^1^·K^−^^1^	962	200
thermal conductivity/W·m^−^^1^·K^−^^1^	92.6	46
expansion coefficient/K^−^^1^	2.06 × 10^−^^5^	4.7 × 10^−^^6^

**Table 3:** The cutting parameters setup of high-speed milling experiment.

Cutting Parameters	Value
Cutting speed	300~1200 m/min
Feed	0.03~0.11 mm/rev
Cutting depth	0.5 mm
Cutting width	3 mm

## Data Availability

The data presented in this study are available in article.
